# Coronary subclavian steal

**DOI:** 10.1007/s12471-023-01772-5

**Published:** 2023-03-30

**Authors:** Jules R. Olsthoorn, Niels Verberkmoes

**Affiliations:** Department of Cardiothoracic Surgery, Catharina Hospital, Eindhoven, The Netherlands

**Fig. 1:**
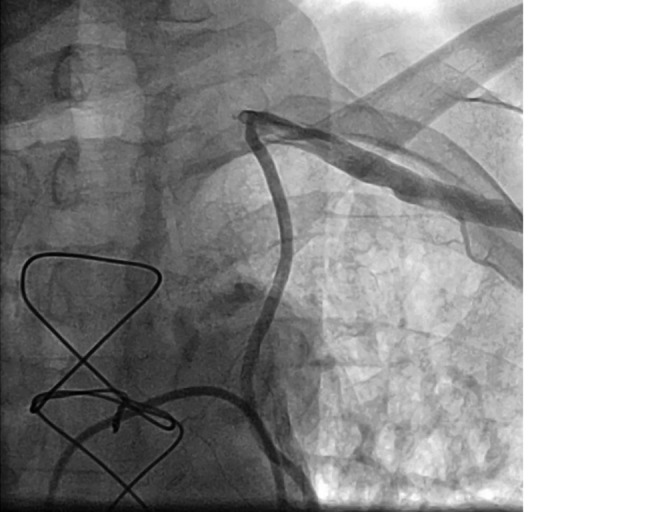
Still frame of postoperative coronary angiography showing retrograde filling of left mammary artery with steal in subclavian artery

Coronary artery bypass grafting (CABG) is the cornerstone of treatment of patients with coronary artery disease [1]. The left internal mammary artery (LIMA) is the gold-standard conduit of choice because of its long-term patency [2]. In patients referred for surgery, atherosclerotic disease is not limited to the coronary arteries and often generalised in the vascular system. The presence of stenosis of the subclavian artery, before the LIMA, is an important problem in patients who have undergone CABG. In 1.0% of cases, this phenomenon occurs postoperatively and can lead to recurrent angina [3]. In extreme cases, as presented in the current report (Fig. 1 and see Video 1 in Electronic Supplementary Material), the flow through the LIMA can invert due to postoperative subclavian occlusion, causing ‘subclavian steal’. In most cases, patients exhibit angina after exercise of the upper extremities. Stenting of the subclavian artery or left subclavian artery bypass can be performed to treat this abnormality.

### Supplementary Information

**Video 1** Postoperative coronary angiography showing retrograde filling of left mammary artery with steal in subclavian artery
